# Multi-UAV Path Planning for Autonomous Missions in Mixed GNSS Coverage Scenarios

**DOI:** 10.3390/s18124188

**Published:** 2018-11-29

**Authors:** Flavia Causa, Giancarmine Fasano, Michele Grassi

**Affiliations:** Department of Industrial Engineering, University of Naples Federico II, Piazzale Tecchio 80, 80125 Naples, Italy; giancarmine.fasano@unina.it (G.F.); michele.grassi@unina.it (M.G.)

**Keywords:** multi-UAV path planning, cooperative navigation, GNSS-challenging environment, insertion-based techniques, polynomial path

## Abstract

This paper presents an algorithm for multi-UAV path planning in scenarios with heterogeneous Global Navigation Satellite Systems (GNSS) coverage. In these environments, cooperative strategies can be effectively exploited when flying in GNSS-challenging conditions, e.g., natural/urban canyons, while the different UAVs can fly as independent systems in the absence of navigation issues (i.e., open sky conditions). These different flight environments are taken into account at path planning level, obtaining a distributed multi-UAV system that autonomously reconfigures itself based on mission needs. Path planning, formulated as a vehicle routing problem, aims at defining smooth and flyable polynomial trajectories, whose time of flight is estimated to guarantee coexistence of different UAVs at the same challenging area. The algorithm is tested in a simulation environment directly derived from a real-world 3D scenario, for variable number of UAVs and waypoints. Its solution and computational cost are compared with optimal planning methods. Results show that the computational burden is almost unaffected by the number of UAVs, and it is compatible with near real time implementation even for a relatively large number of waypoints. The provided solution takes full advantage from the available flight resources, reducing mission time for a given set of waypoints and for increasing UAV number.

## 1. Introduction

Recently, an increasing effort has been concentrated on low altitude small Unmanned Aircraft System (UAS) applications such as small packet delivery, urban surveillance, infrastructure inspection, and three-dimensional mapping [1,2]. In these scenarios, reliable flight autonomy is one of the key capabilities to unleash the potential of UAS and maximize their advantages. In general, the concept of autonomy may involve all mission phases, from automated flight planning (and re-planning, if necessary) to real time guidance, navigation, and control. In this framework, several mission profiles may require the unmanned aircraft to fly in heterogeneous environments, comprising both areas with nominal Global Navigation Satellite Systems (GNSS) coverage, and challenging or denied environments such as natural or urban canyons.

When flying under nominal satellite coverage, navigation performance achieved by standalone or differential GNSS/inertial technology typically fulfills flight needs. On the other hand, in challenging scenarios autonomous flight is hindered by navigation issues. Navigation in challenging environments is usually tackled by using additional measurements, i.e., visual-aided navigation [3], radio beacon [4,5], positioning based on phone signals [6] and actuator outputs [7]. A promising strategy to compensate for the lack of reliable GNSS measurements is represented by cooperative navigation [8]. Cooperative or networked navigation is a term used to describe an approach whereby a community of users exploits shared measurements and information exchange to the navigation advantage [9,10,11]. In general, its implementation requires a multi-UAV architecture and may gain advantage from proper flight geometries [12,13]. In this framework, the authors proposed in previous works [13,14,15] an approach that exploits information broadcast and relative sensing, where one or more vehicles flying in areas not susceptible to GNSS signal corruption, designed as “father” UAV(s), are used to support autonomous navigation of a “son” UAV flying in challenging conditions, by broadcasting their positioning information and acting as visual features/trackers and/or transponders for radio frequency-based ranging. 

**Figure 1:**
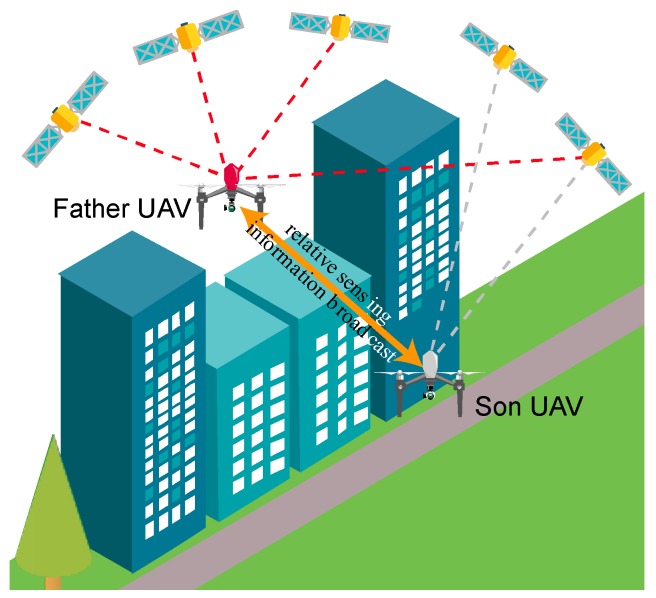
Conceptual view of GNSS challenging area, the son (gray) has a non-nominal GNSS coverage (less than four satellites) and is inside the GNSS challenging area. The father UAV (Red) always holds a line-of-sight link with the son, while remaining outside the challenging area.

Reference [13] shows that positioning accuracy for the son UAV depends on several factors, including father-son and GNSS coverage geometry. However, the basic requirements that the father has to fulfill consist in keeping an unobstructed line-of-sight to the son, and flying under good satellite coverage (i.e., outside of the challenging areas) not too far from the son. Father(s) and son UAVs have to fly in a coordinated way to ensure success of the cooperative navigation strategy. A conceptual view of the cooperation concept is shown in Figure 1. As shown in [15] for a tandem formation (one father one son), when an entire mission has to be carried out in a challenging environment (e.g., 3D mapping in a canyon), father and son UAVs keep their role during the whole mission. However, it is intuitive that this choice is non-optimal in scenarios with heterogeneous GNSS coverage conditions. In fact, coordinated flight and father/son task assignment is of interest only in some phases of the mission, while the different UAVs can be used as independent systems in absence of navigation issues. Thus, in these scenarios, the need to optimize the usage of available resources to minimize mission time while enhancing flight autonomy, naturally leads to an integrated approach to multi-UAV path planning, guidance, and navigation.

This paper focuses on multi-UAV path planning for these mixed GNSS coverage scenarios. It aims at defining flyable trajectories for a swarm of UAVs, whose purpose is to reach several waypoints (also defined as “targets” in the following) and then performing actions at each location. Targets can lie in challenging or non-challenging areas. The proposed problem can be formulated as a customization of the vehicle routing problem (VRP) [16] that must account not only for target distribution amongst UAVs, but also on the need to ensure autonomous flight in challenging areas, associating to each son the needed number of fathers. The proposed approach can be thus defined as “navigation aware” path planning for multiple UAVs. The result is a multi-UAV system that is able to reconfigure itself during a mission, based on the operating environment. 

In the open literature, “navigation aware” planning techniques for GNSS challenging environments are aimed at defining trajectories that minimize the state covariance [17,18,19], or that ensure a collision free path in the limits of the available GNSS measurements [4,20]. These approaches adapt the trajectories not only to the mission requirement, but also account for the navigation state, which limits the areas the UAVs can reach. Exploiting cooperation, the approach proposed in this paper aims at defining trajectories that pass through mission defined targets, without forcing the trajectory of the vehicles to be modified because of navigation needs (in GNSS challenging areas), but using father(s) to reduce navigation state estimation covariance.

Several authors in the open literature have focused on the problem of vehicle routing for multiple UAVs. The available strategies for VRP can be divided in exact and heuristic or metaheuristic methods [21,22]. Exact resolution problems [23,24,25] are formulated as mixed-integer linear programming (MILP), and solved with branch and bound or set covering techniques [16]. These approaches yield the optimal solution by exploring all the feasible combinations, which suffers from scalability issues requiring a significant computational burden for increasing number of UAVs and/or target waypoints. In addition, using exact resolution methods requires formulating the problem as linear, which becomes complicated when the “classic” VRP is customized with additional constraints, as in the considered scenario. When this occurs different heuristic [26] or metaheuristic [26,27,28] approaches can be applied. Bertuccelli et al. and Moon et al. [29,30] use consensus based bundle algorithm to route a swarm of UAV in cluttered environment. Genetic algorithm and a discrete version of the particle swarm optimization (PSO), are used for assigning targets to UAVs in [31] and [32], respectively. In this paper we use an iterative heuristic method, i.e., the insertion-based algorithm [33,34], since the use of heuristics reduces the computation time, whilst, the sequential approach allows controlling which type of target is to be assigned after each iteration. As an example, the proposed algorithm assumes that after assignment of targets in a challenging area, the subsequent target to be assigned is a father one. Father locations (targets) are not defined *a priori*, but during target assignment, that makes unsuitable the usage of techniques that do not allow input data (i.e., targets) changing, e.g., PSO, MILP or genetic algorithm. 

**Figure 2:**
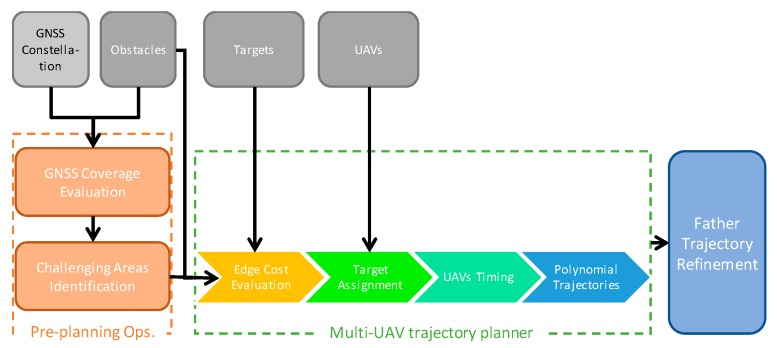
Scheme of path planning for a swarm of UAVs in a challenging environment, this paper tackles the central step of the scheme termed as “Multi-UAV trajectory Planner”, while “Father Trajectory Refinement” is analyzed in [15], and details about pre-planning operations are given in [13] and summarized in Section 2.

To ensure that each son is assisted by one or more fathers during the flight in its challenging zone, the VRP solution is then complemented with a proper timing strategy. Finally, flyable smooth paths are defined as polynomial trajectories based on the waypoint sequences and the timing solution. More in general, the conceived framework for autonomous multi-UAV missions in complex heterogeneous environments is depicted in Figure 2. It includes:

- Preprocessing operations aimed at evaluating the GNSS-challenging zones where UAVs cannot fly autonomously without support from other vehicles. They are based on the knowledge of a coarse representation of the 3D mission environment, and of the time and date the mission is performed (i.e., GNSS constellation geometry). Once the constellation of satellites and the surrounding environment are known, it is possible to define a 3D grid of the volume where the UAVs are designed to fly, in order to map the dilution of precision (DOP) and define the challenging areas as explained in Section 2;
- A multi-step path planning algorithm that assigns to all the UAVs flyable trajectories that fulfill mission and navigation constraints. This is discussed in detail in this paper;
- An algorithm to refine the trajectories of the father UAVs when operating in challenging zones. This algorithm allows, once the trajectories of the sons in challenging areas are defined, shaping father trajectories based on the navigation needs. This is oriented towards real time guidance and is described in [15].

The main contribution of the paper is connected to the integration of cooperative navigation at planning level, thus enabling optimization of resources and autonomous flight through mission-defined targets in spite of navigation issues. The main innovation of the planning approach proposed in this paper is the introduction of a novel technique that solves together the problems of vehicle routing, task (father/son) assignment in challenging areas, and cooperative timing to ensure that father and son operate in the same challenging area at the same time. It uses a customized 3D fast insertion-based task assignment algorithm, with an adaptive cost function that is aimed at minimizing the total path length while also ensuring uniformity in load distribution amongst UAVs. Moreover, additional waypoints are defined during the planning process to account for cooperative navigation needs. Finally, it introduces an original timing strategy, that synchronizes UAVs motion accounting for the different speeds achievable in challenging areas or in open sky conditions.

The paper is organized as follows: Section 2 introduces the developed planning concept, its assumptions and the main processing steps. Algorithms are then detailed in Section 3, Section 4, Section 5 and Section 6. Performance assessment is discussed in Section 7, where path planning is tested in a real-world scenario in the city of Naples. Finally, concluding remarks and future research directions are presented in Section 8. 

## 2. Planning Concept and Assumptions

The trajectory planning algorithm described in this paper assumes that waypoints (targets) and service time, i.e., the time needed to perform the demanded mission at each waypoint, are provided as input data. Indeed, their definition usually depends on the specific mission under analysis and the adopted payload (e.g., infrastructure inspection [35]). In general, input data for the assumed multi-UAV path planning in heterogeneous environment include:

- A sequence of target positions, with the associated service time;
- Definition of GNSS-challenging areas (where navigation requirements cannot be fulfilled by single vehicle techniques), and of the number of fathers required at each of them for supporting the son flight. This information is the output of pre-processing operations based on a coarse knowledge of geometry of the three-dimensional (3D) environment (including obstacles), the GNSS geometry at the time the mission must be executed, and the assumed cooperative navigation sensors/approaches [13];
- Definition of eventual no-fly zones, which are seen as obstacles;
- Number and dynamic constraints (e.g., maximum speed) of the adopted UAVs. It is assumed that all the UAVs have the same constraints and capabilities.

The definition of the challenging zones is performed analyzing which are the areas where GNSS satellites in view are not able to guarantee a certain navigation error. In those areas, the number of father vehicles depends on the available GNSS information and the adopted cooperative sensors. As a general concept, in GNSS-challenging areas, cooperative measurements are needed to provide given bounds for positioning error, complementing (eventual) pseudorange information from GNSS satellites. Available pseudorange information depends on the three-dimensional environment and the current GNSS coverage. Cooperative aiding measurements depend on which systems are adopted for relative sensing: cameras [36] (on board father or son) and/or RF-ranging systems can be used, that provide angular and/or range information. From a practical point of view, at least four scalar measurements are needed to bound the positioning error. As an example, if the son has only two GNSS satellites in view, and RF-ranging is used for relative sensing, then two fathers are needed. In the same GNSS coverage conditions, a single father equipped with a camera (providing two angular measurements) can be able to fulfill navigation needs and bound the positioning error. It is clear that the quality of cooperative measurements, and the geometry of the problem, strongly impact the available positioning performance, which can be described by the concept of “generalized dilution of precision” [13]. 

For the sake of simplicity, this paper assumes that both obstacles and challenging areas are prisms, and that variations in terrain elevation can be neglected. If there are no targets in a specific challenging area, that area is seen as an obstacle. It could be noticed that passing through a challenging area, instead of finding an avoidance path, could bring in theory an advantage in terms of mission time minimization. However, since father support is needed, then, the velocity of the son should be small enough to ensure father-son line of sight link to be always available, and one or more UAVs are needed for cooperation. Thus, even if the distance covered by the son to pass through the challenging area is lower than the one needed to avoid that zone, the overall mission time may be increased due to the reduced velocity and the need to use more than one UAV to pass that area. In addition, given the challenges of son-father formation flight, our approach is thus to fly UAVs in challenging zones only if this is required by the mission. On the other hand, if the challenging area includes at least a target, only a single UAV, designed as son for that area, is allowed to access it. All the other UAVs see the considered area as a no-fly zone. This choice is made to avoid congestion in areas that are characterized by navigation challenges.

The start and the end point of the trajectory are the same for each UAV, and it is assumed that at *t* = 0 all the UAVs are at the start location. The velocity that son UAVs should not exceed during their flight in the challenging areas is defined as *v_chall_*, while the cruise velocity, that is the one that should not be exceeded outside challenging zones, is named *v_cruise_*. Due to the lack of reliable GNSS coverage and the need of maintaining unobstructed line-of-sight with the father(s), it is reasonable to assume that *v_cha_*_ll_ is relatively small, about 20–30% of the cruise velocity *v_cruise_*.

Given this input information and assumptions, routing vehicles basically requires each selected target to be assigned to one UAV. Furthermore, in addition to classic VRP scenarios, when a vehicle flies inside a GNSS-challenging area (i.e., it is deemed as son for that area), one or more UAVs (father UAVs for that challenging area) need to “serve” the son supporting its flight by relative sensing and information broadcast. Therefore, planning is not limited to assign targets (waypoints) to each vehicle but must include a strategy to define, for each challenging zone, father UAV(s) and the associated waypoints. As far as this paper is concerned, the definition of father waypoints is aimed at fulfilling the basic requirements recalled in the introduction (i.e., unobstructed LOS to the son, flight outside challenging areas at reduced distance from the son). Father trajectories can then be optimized in a refinement phase [15], which has negligible impact at planning level.

The strategy used for assigning targets consist in optimizing the resources (UAVs) to reduce the time of mission completion. Reducing mission time means reducing the overall distance covered by the UAVs and in addition equalizing the path length amongst the UAVs, to ensure the paths have more or less the same duration. Figure 2 shows the main steps of the path planner that are detailed in the following sections.

- The first step (edge cost evaluation, presented in Section 3) is aimed at defining obstacle-free paths between each couple of targets, and evaluating their length;
- The second part of the path planning algorithm (target assignment, Section 4) assigns all the waypoints and tasks to the UAVs, with the aim of minimizing the overall mission time. This is done minimizing the total path length while also ensuring uniformity in load distribution among UAVs. As an output of this step, each UAV is assigned a trajectory that is a polygonal chain composed by a number of waypoints and the edges between them;
- The timing step of the planning algorithm (Section 5) consists in defining the velocity that each UAV must hold along its trajectory in order to synchronize son and father arrival and departure to/from the challenging zones;
- Finally, polynomial paths are defined for all the UAVs to connect waypoints with flyable and smooth trajectories (Section 6). Polynomials allow easily deriving 3D position and its derivatives for each time epoch.

## 3. Edge Cost Evaluation

Edge cost evaluation is aimed at estimating the cost to travel along each possible edge, i.e., the piece of trajectory between each couple of the available waypoints. For this specific application, the edge cost for each couple of targets *i* and *j* can be estimated as the length of the path between them, defined as *d_ij_*. For the sake of edge cost evaluation, the path between two targets can be thought as an obstacle-free polygonal chain, with its length estimated as the sum of the lengths of its segments. 

**Figure 3:**
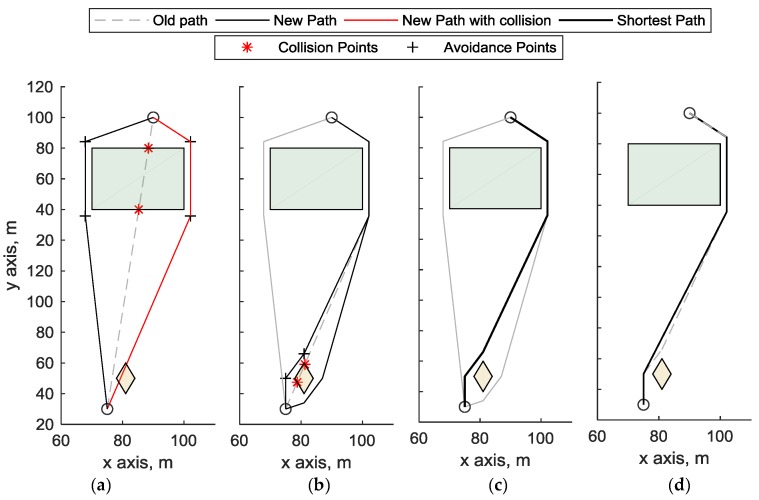
Definition of obstacle-free path between two waypoints. (**a**) avoidance of green obstacle, on the top. Two avoidance paths are depicted, the black one is obstacle-free, the red one intersects the bottom obstacle; (**b**) avoidance of the orange (bottom) obstacle: the two avoidance paths (in black) are not intersecting any other obstacle; (**c**) Among the three non-colliding paths that connects the two waypoints (circles) the one with the minimum path length (black) is selected. (**d**) Fibonacci filtering is applied to remove the unneeded point from the selected trajectory.

A multi-step process is adopted to obtain obstacle-free paths. The path between waypoints is initially defined as the straight segment that connects them. If the segment intersects an obstacle, auxiliary points are generated in proximity of obstacle corners. Different polygonal chains are thus obtained. These are then re-checked for obstacle avoidance, and further auxiliary points may have to be added. When all the potential paths are obstacle-free, the shortest one is selected, and a Fibonacci filter [30] is used to remove unneeded nodes. Within edge cost evaluation, challenging zones are considered as no-fly zones (i.e., an obstacle), when both targets *i* and *j* lie outside that area. The procedure is shown in Figure 3. The straight-line path between the two waypoints (indicated as circles) intersects the green obstacle on the top, and the two avoidance paths are defined in Figure 3a. The avoidance paths are defined as the paths that travel around the obstacle passing through avoidance points (black crosses), that are points located at 3 meters along the bisector of each corner. One of the so defined paths (highlighted in red in Figure 3a) still intersects the bottom obstacle, therefore two new paths avoiding the bottom obstacle are computed in Figure 3b. Figure 3c shows all the generated obstacle-free paths that connect the two waypoints, where the black is the one with minimum length. Figure 3d shows the Fibonacci filter application to the path with the minimum length, which removes an unneeded point from the trajectory.

## 4. Target Assignment

**Figure 4:**
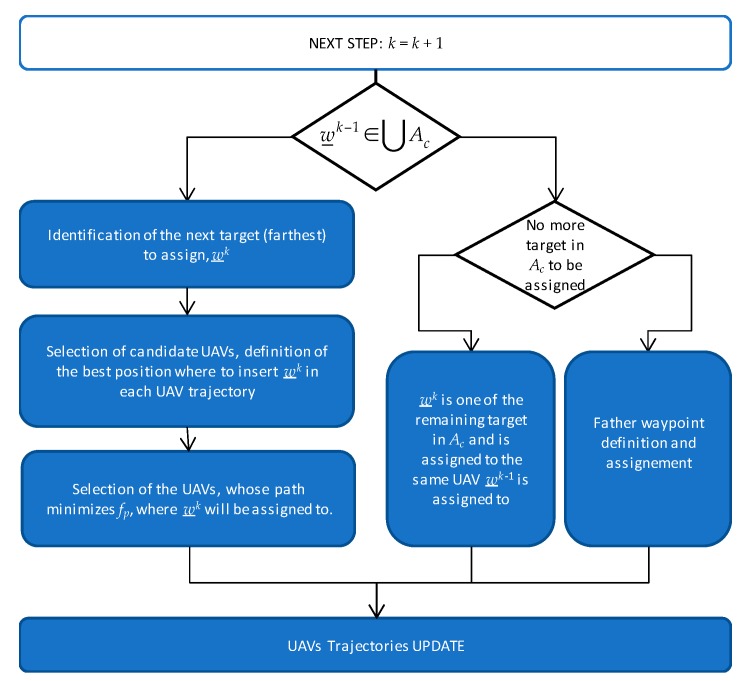
Target Assignment algorithm.

The insertion-based algorithm used to sequentially assign target to the trajectory aims at optimal distributing the resources amongst the targets. Let *n* be the number of targets, *m* the number of available UAVs, *A_c_* the *c*-th challenging zone, with c=1,…,C. The targets to be assigned are named ${\underline{w}}_{i}=1,\ldots ,n$, and include the start and the end waypoints, that are common to all the UAVs. The waypoints that are sequentially assigned to the trajectory are indexed with an apex *k*, i.e., *w*^k^, that indicates the step of the task assignment algorithm at which they are assigned. The assignment sequence is not known *a priori*. The target/task assignment algorithm at step *k* is shown in Figure 4. 

There are three possible cases. If the waypoint assigned at the previous step (*w^k^*^−1^) lies within a challenging area *A_c_*, and no other waypoints in that area need to be assigned, the targets to be assigned at step *k* are father waypoints, whose definition and assignment procedure is reported in Section 4.2. If *w^k^*^−1^ lies within a challenging area *A_c_* and other waypoints lie in the same challenging zone which have not been assigned yet, these other waypoints are assigned to the UAV already designated as son for that zone. Finally, if *w^k^*^−1^ does not belong to any challenging area, the waypoint insertion procedure described in Section 4.1 is applied.

### 4.1. Waypoint Insertion Procedure

Let $p_{h}^{k-1}\left(w_{h}^{k-1},e_{h}^{k-1}\right)$ be the trajectory of *h*-th UAV at step *k* − 1 that includes a set of waypoints (**w**) and edges (**e**) currently assigned to that UAV. The sorted sequence of waypoints assigned to the *h*-th UAV at step *k* − 1 can be written as $w_{h}^{k-1}=\left[\begin{array}{ccc} {\underline{w}}_{h}^{1} & \ldots & {\underline{w}}_{h}^{h_{k-1}} \end{array}\right]$, where $h_{k-1}$ is the number of waypoints assigned to that UAV at step *k −* 1. At step zero, each UAV trajectory includes only the start and the end waypoints of the trajectory and the edge defined in between them. The waypoint insertion procedure consists in choosing the target to be added to the trajectory set, and selecting the UAV to which this waypoint must be assigned to. It is based on minimizing a proper cost function. At each step, the target to be assigned is the “farthest” target, defined as the target that maximizes the distance from all the assigned waypoints and edges, while the cost function aims at minimizing the sum of path lengths for the different UAVs and keeping uniformity in the path length distribution. As it will be shown in the following, the assignment of the farthest target to the UAV with the minimum path length increase is a strategy that mimics optimal approaches such as MILP. In details, waypoints insertion procedure consists in the following three steps, also summarized in Figure 4:

(a) Select the target *i* to be added (“farthest” target) as the one that maximizes *f_T_*:
  (1) $$f_{T}=\frac{1}{J}\sum_{j=1}^{J}d_{ij}+\frac{1}{L}\sum_{l=1}^{L}D_{il},$$
  where *d_ij_* is the distance (computed along the relevant edge) between the not yet assigned target *i*, and the already assigned target *j*. *j* enumerates the already assigned waypoints (including fathers’ ones) and *l* the already assigned edges, whose distance from the target *i* is named *D_il_*.
(b) Find the three edges that are closest to the farthest target, and the UAVs whose trajectories at *k*-1 include at least one of the endpoints (*w_e_*) of these edges. For each UAV, the farthest target is tried to be inserted before and after the point *w_e_*. The resulting paths that intersect the path of the other UAVs are discarded to avoid that targets could be assigned to farther trajectories when the path equalizing logic prevails. In addition, this improves the capability of the algorithm to mimic optimal techniques. Then, the best insertion location is defined as the one that minimizes path increase. The trajectory obtained by adding the farthest target to the path of the *g*-th UAV is defined as ${\overset{⌣}{p}}_{g}^{k}\left({\overset{⌣}{w}}_{g}^{k},{\overset{⌣}{e}}_{g}^{k}\right)$
(c) The UAV which the target is assigned to, is selected minimizing the cost function *f_p_*, reported in Equation (2). This cost function is composed by two terms aimed at minimizing the overall distance and reducing the standard deviation (std) among UAV path lengths, thus ensuring (up to a certain level) uniformity in load distribution among UAVs. This is an innovative point of the target assignment procedure. The cost function is written as:
  (2) $$f_{p}=\frac{\frac{1}{\alpha }\mathrm{std}\left(\left[d\left(P^{k-1}/p_{g}^{k-1}\right);d\left({\overset{⌣}{p}}_{g}^{k}\right)\right]\right)+\alpha \cdot \left(d\left({\overset{⌣}{p}}_{g}^{k}\right)-d\left(p_{g}^{k-1}\right)\right)}{\mathrm{mean}\left(\left[d\left(P^{k-1}/p_{g}^{k-1}\right);d\left({\overset{⌣}{p}}_{g}^{k}\right)\right]\right)},$$
  where $d\left(P^{k-1}/p_{g}^{k-1}\right)$ is the vector containing path lengths at step *k*-1 for all UAVs excluding the *g*-th UAV, for which path length is computed accounting for the farthest target added at the *k*-th step and defined as $d\left({\overset{⌣}{p}}_{g}^{k}\right)$. $d\left(p_{g}^{k-1}\right)$ is the path length of the *g*-th UAV at time step *k* − 1. *mean* is the operator that yields the mean of the variables, *α* is a tuning coefficient whose role is relevant to the trade-off between path lengths uniformity and minimization.

In fact, the first term at the numerator is the standard deviation of the path lengths and is used to make as uniform as possible the distribution of path lengths among trajectories. The second term at numerator aims at assigning the waypoint to the UAV that has the minimum path increase after the waypoint is added to its trajectory and is thus aimed at minimization of total path length. These two elements are weighted by the coefficient *α* that is small at the first and last steps of assignment procedure, where the logic that prevails is to make trajectories as uniform as possible. 

In the central steps of assignment procedure, *α* is higher and the aim of assignment procedure is biased towards minimization of the overall distance to be covered. *α* is a quadratic function of *k* and it is equal to *α_max_*, when *k* = *n*/2 and equal to *α_min_* when *k* is 1 or *m. α_min_* and *α_max_* are set by the user. 

**Figure 5:**
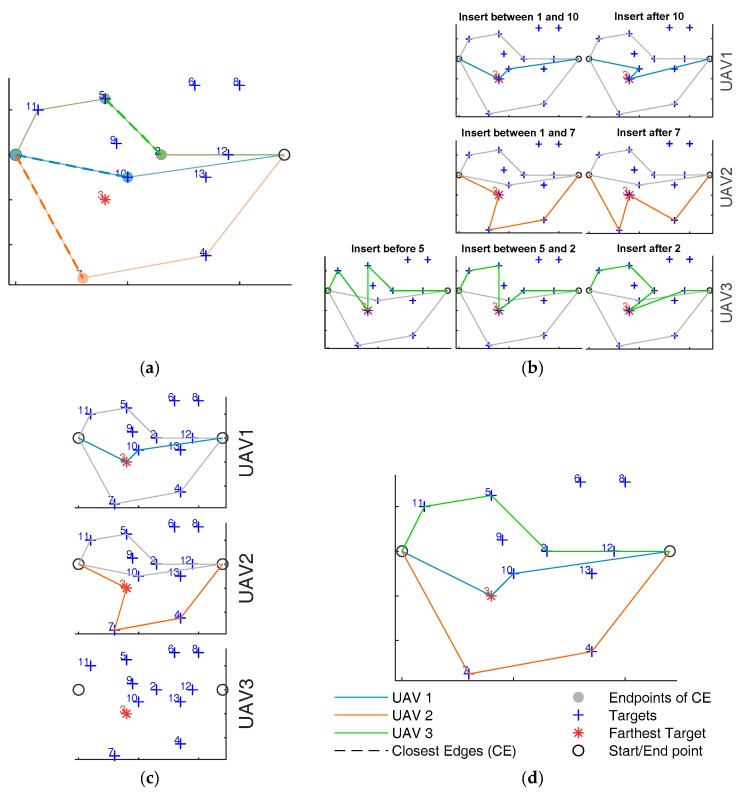
Example of waypoint insertion procedure with 1 target and 3 UAVs (*n* = 14 and *m* = 3). (**a**) selection of the farthest target and definition of the three closest edges and their endpoints *w*_e_, (**b**) Insertion of the farthest target before and after *w*_e_, (**c**) identification of the shortest path for each UAV, neglecting the paths that intersect those of the other UAVs. (**d**) Assignment of the target to the UAV whose path minimizes the cost function *f_p_* in Equation (2).

Figure 5 shows an example of waypoint assignment procedure applied for the sake of simplicity at 2D scenario (i.e., the altitude of the target is the same) with 14 targets and 3 UAVs (*n* = 14 and *m* = 3). In the case depicted in the figure, seven targets (2-4-5-7-10-11-12) have already been added to the trajectory, thus *k* = 8. The farthest target at this step is the waypoint 3, and the three closest edges are 1-10, 1-7, 5-2, that belong to the current trajectories of UAV 1, UAV 2 and UAV 3, respectively. In Figure 5b the farthest target is tried to be inserted before and after each endpoint of the three closest edges, resulting in an increment of the path of the UAV where the endpoint belongs. Figure 5b depicts the possible insertions for the three UAVs. For each UAV the unfeasible paths (those that intersect the trajectory of the others) are removed. In the specific case in the figure, all the paths of UAV 3 must be removed since they intersect the already defined path of UAV 2. Then the shortest path is estimated for each UAV, as in Figure 5c. Figure 5d represents the final trajectory after the insertion of the farthest target to the UAV, which path minimizes Equation (2) (UAV 1 in the considered example). 

### 4.2. Father Waypoint Definition and Assignment Procedure

**Figure 6:**
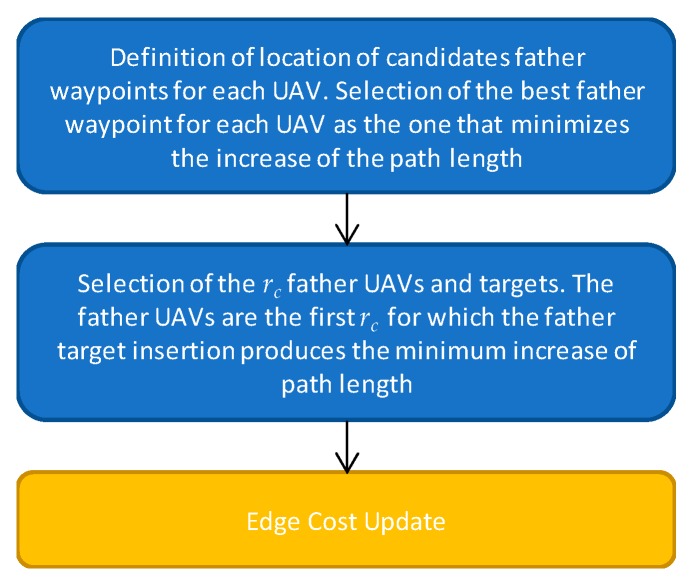
Father Waypoint Definition and Assignment procedure.

As anticipated above, when more than one target is inside a challenging area, all these targets are assigned to the same UAV that is designed as the son UAV for that zone. When the target assigned at step *k* − 1 lies in a specific challenging zone *A_c_* and no other targets in that zone need to be assigned, the next step consists in defining father waypoints for that challenging zone, and assigning them to specific UAVs. These are auxiliary waypoints, not foreseen in the initial targets definition and directly related to navigation needs. The basic input information from cooperative navigation approaches concerns the number of father UAVs required for a given zone. As in other processing steps, definition and assignment of father waypoints is done according to path length minimization principles. The main steps that compose fathers’ assignment strategy are summarized in Figure 6, and described in the following:

(a) For the *c*-th challenging zone, candidate father waypoints $x_{c}=\left[\begin{array}{ccc} {\underline{x}}_{c}^{1} & \ldots & {\underline{x}}_{c}^{o_{c}} \end{array}\right]$, are estimated assuming that father(s) can be placed on an open face of the *c*-th challenging volume, where *o_c_* is the number of open faces of that volume. Since that volume is a prism, one can easily identify the open faces as the ones not adjacent to any obstacle. For each open face, the candidate waypoint is defined projecting the barycenter of the targets inside the challenging area on a plane parallel to the face and located at a distance of 3 m from it (outside the challenging zone). It is assumed that the UAV designed as father must hold that position for the whole time required to the son UAV for flying inside the challenging zone, unless the father target is located on top of the challenging volume. In that case the father UAV flies over the challenging area passing by the father waypoints.
  Candidate father UAVs are all the UAVs, excluding the one that is son for the *c*-th challenging zone. For each candidate UAV, all the possible father waypoints are tried to be inserted in between all the waypoints belonging to the trajectory at step *k* − 1. The best insertion is defined, along with the best father, as the couple that minimizes the increase of path length $\Delta_{c,i_{c}}^{h,i_{h}}$:
  (3) $$\begin{array}{c} \delta_{\min,h}=\underset{i_{h},i_{c}}{\min}\Delta_{c,i_{c}}^{h,i_{h}}\\ \Delta_{c,i_{c}}^{h,i_{h}}=\Delta w_{c,i_{c}}^{h,i_{h}}+\Delta_{v_{c}}\\ \Delta w_{c,i_{c}}^{h,i_{h}}=\left\|{\underline{w}}_{h}^{i_{h}+1}-{\underline{w}}_{c}^{i_{c}}\right\|+\left\|{\underline{w}}_{c}^{i_{c}}-{\underline{w}}_{h}^{i_{h}}\right\|-\left\|{\underline{w}}_{h}^{i_{h}+1}-{\underline{w}}_{h}^{i_{h}}\right\| \end{array}$$
  where $\Delta_{v_{c}}$ is the increase of the *h*-th UAV trajectory length due to adding *i_c_* father point after the *i_h_* element of the ${\underline{w}}_{h}^{k-1}$ sequence. ‖ ‖ is the Euclidean distance, used instead of the distance on the obstacle-free polygonal chain, to simplify operations and reduce the computational burden. $\Delta_{v_{c}}$ is the total distance between the targets served by the son UAV in the *c*-th challenging zone, that is summed up to the Euclidean increase of trajectory length. $\Delta_{v_{c}}$ is added to $\Delta w_{c,i_{c}}^{h,i_{h}}$, to take into account the fact that the father trajectory must be defined to serve the son UAV within the challenging zone, and the time spent to do this is strictly connected with the time the son UAV requires to fly inside the challenging area. Indeed, when planning the father trajectory, one must account for the time spent to serve the son, when the father must fly over or hover next to the challenging area.
(b) The UAVs to which father targets are assigned are the first *r_c_* for which $\delta_{\min,h}$ is smaller, where *r_c_* is the number of required fathers for the *c*-th challenging zone. If more than one UAV choose the same father position, i.e., the same face from where to serve the son, evenly spaced points around the initially considered father position are designed as UAV father points to prevent those UAVs from holding the same position during son operations. Therefore, father assignment yields *r_c_* new points to the UAVs trajectory, even if some of the fathers serve the son using the same face. As previously pointed out, father waypoints are only an indicative location for the true father trajectory in servicing the son. The definition of the specific father/son aiding geometry can be left to cooperative navigation studies [15], while the presented definition of father waypoints has sufficient level of detail in view of path planning aims.
(c) The last step consists in updating the edge cost definition including the *r_c_* father waypoints. The cost to travel from the newly defined father targets to the already defined *w_i_* targets is estimated, in order to account also for the father waypoints in the definition and assignment of the farthest target for the next steps of the assignment procedure.

## 5. UAV Timing

**Figure 7:**
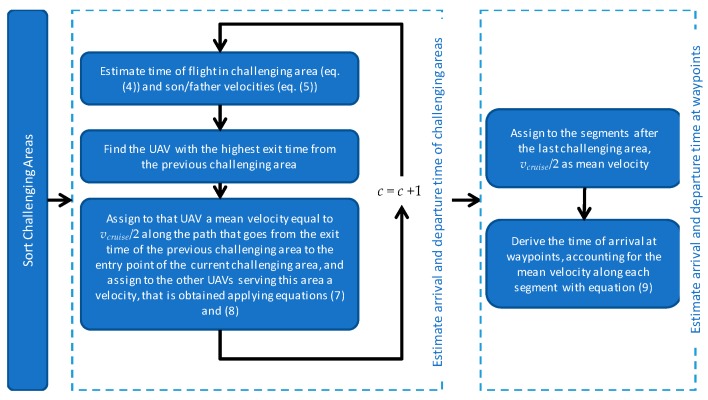
UAV Timing Strategy.

The previous processing steps define son and father(s) that operate in each challenging zone. UAV Timing, whose flow chart is depicted in Figure 7, defines the time the UAVs arrive and depart from a certain location (target) so that father and son arrive and leave the challenging zones at the same time. UAV timing can be divided in two steps. The first described in Section 5.1 that yields the time of arrival and departure of the UAVs from the challenging areas. The second (Section 5.2) is aimed at defining the time of arrival and departure of each UAV at any waypoint of its trajectory.

### 5.1. Arrival and Exit Time of the Challenging Areas

**Figure 8:**
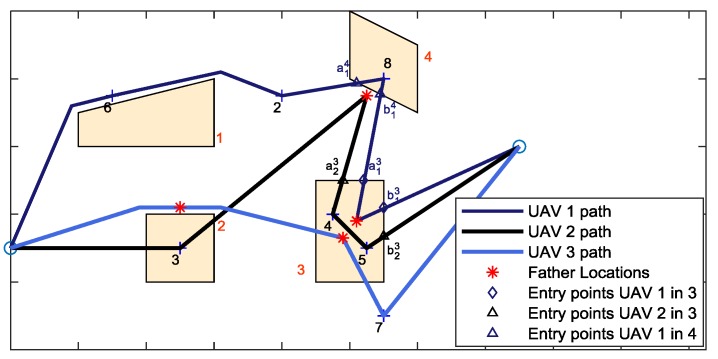
Path of three UAV in performing mission in an environment with heterogeneous GNSS coverage (horizontal view). Orange areas are GNSS challenging zones where cooperative navigation is required. The figure shows the distribution of the target amongst the UAV. The father for challenging area 2 is UAV 3, that is serving the son laterally. The fathers for challenging area 3 are UAVs 1 and 3 that fly above that zone, whilst UAV 2 is father for challenging area 4.

Assignment of the required time to fly from one challenging area to the following one occurs sequentially. Thus, the challenging areas are sorted based on the order in which they are served by the UAVs. Operation time in each challenging area depends on son UAV parameters, such as path length in the challenging zone and service time at each target. Instead, the time of arrival at the challenging area depends on the exit time from the previous challenging areas. Especially in complex scenarios with several challenging zones, it is likely that the UAVs serving the *c*-th challenging area are coming from different challenging areas, with different exit times. As an example, in Figure 8 all three available UAVs used for the mission are needed to support the flight in challenging area 3. Although the previous challenging area both for UAV 1 and UAV 2 is the area number 4, the UAV 3 comes from area number 3. 

The arrival time is evaluated as follows. First, the UAV with the maximum exit time from the previous challenging areas is considered. This UAV is assigned an average velocity along the path from the previous to the current challenging area, which defines its arrival time. Then, velocities for the other UAVs operating at the challenging area are evaluated imposing that all the arrival times should be the same. If one of these velocities exceed the dynamic capabilities of the aircraft, it is set equal to the UAV maximum speed, and both the arrival time and the average velocity of the other UAVs are updated. 

In details, the time of flight of the son UAV in the *c*-th challenging area, and thus the time of father(s) aiding for that area, is equal to:

(4) $$\Delta t_{c}=d\left(p\left({\underline{a}}_{S}^{c}:{\underline{b}}_{S}^{c}\right)\right)\frac{2}{v_{chall}}+t_{w}\left({\underline{a}}_{S}^{c}:{\underline{b}}_{S}^{c}\right),$$

where $p\left({\underline{a}}_{h}^{c}:{\underline{b}}_{h}^{c}\right)$ is the path of the *h*-th UAV from the entry point (${\underline{a}}_{h}^{c}$) to the exit point (${\underline{b}}_{h}^{c}$) of the *c*-th challenging area, and d( ) is the length of this path. Δ*t_c_* is obtained by summing up the time required to cover the path inside the zone and the servicing time of the waypoints inside the challenging zone, i.e., $t_{w}\left({\underline{a}}_{S}^{c}:{\underline{b}}_{S}^{c}\right)$. Indeed, each waypoint is related to a servicing time that is the time required for the UAV to perform operation on that target, e.g., acquiring remote sensing data, performing surveillance related operations, carrying out delivery and/or pickup. 

To estimate the flight time of the son in the challenging area, the overall distance of the son in that area is divided by *v_chall_*/2, which is selected as mean velocity to guarantee that despite the velocity variations (e.g., in proximity of the targets, if the son must stop) the maximum velocity of the son in the challenging zone is not greater than *v_chall_*. In summary, the mean velocities of the father(s) and the son in the challenging area are:

(5) $$\begin{array}{c} {\bar{v}}_{S}^{c}=v_{chall}/2\\ {\bar{v}}_{F}^{c}=\frac{d\left(p\left({\underline{a}}_{F}^{c}:{\underline{b}}_{F}^{c}\right)\right)}{\Delta t_{c}} \end{array}$$

In general, father does not enter the GNSS challenging area. When the father flies above the challenging area, its “entry” and “exit” points (i.e., ${\underline{a}}_{F}^{c}$ and ${\underline{b}}_{F}^{c}$) are points whose *x* and *y* coordinates are given by the intersection of the horizontal projections of father trajectory and the top face of the challenging zone, while the vertical coordinate is given by selecting along the father path the point with those *x* and *y* coordinates. In the case the father location is lateral to the challenging area, no entry and exit points exist for the father that must hover in its location waiting for the son (thus, ${\bar{v}}_{F}^{c}=0$). For the sake of clarity, Figure 8 shows the path of three UAVs, whose flights intersect a challenging area. UAV 1 and UAV 2 play the role of fathers for challenging zones 3 and 4, respectively, where their father waypoints are above and lateral with respect to the challenging area. With reference to challenging area 4, UAV 2 hovers at its father location when UAV 1 covers the path from ${\underline{a}}_{1}^{4}$ to ${\underline{b}}_{1}^{4}$. Instead, in the challenging zone 3 the father, i.e., UAV 1, moves from ${\underline{a}}_{1}^{3}$ to ${\underline{b}}_{1}^{3}$.

The time required by the son to fly in the challenging zone, i.e., Δ*t_c_*, connects the exit and entry time of the *h*-th UAV in the *c*-th challenging zone, respectively ($t\left({\underline{b}}_{h}^{c}\right)$ and $t\left({\underline{a}}_{h}^{c}\right)$, respectively):

(6) $$t\left({\underline{b}}_{h}^{c}\right)=t\left({\underline{a}}_{h}^{c}\right)+\Delta t_{c}.$$

Equations (4) and (6) guarantee that father and son will be at the same time at the exit point of the challenging area, if they are at the same time in the entry points. As stated above, to ensure the entry time in the challenging zone is the same for father(s) and son vehicle, one must account for the paths those UAVs have covered before arriving in that area. 

Let h=1,…,H be the index defining the UAVs that serve the *c*-th challenging zone as fathers and son. The arrival time of the *h*-th UAV at ${\underline{a}}_{h}^{c}$ is:

(7) $$t\left({\underline{a}}_{h}^{c}\right)=t\left({\underline{b}}_{h}^{c-1}\right)+\frac{\bar{v}\left(p\left({\underline{b}}_{h}^{c-1}:{\underline{a}}_{h}^{c}\right)\right)}{d\left(p\left({\underline{b}}_{h}^{c-1}:{\underline{a}}_{h}^{c}\right)\right)}+t_{w}\left({\underline{b}}_{h}^{c-1}:{\underline{a}}_{h}^{c}\right),$$

where $t\left({\underline{b}}_{h}^{c-1}\right)$ is the exit time of the *h*-th UAV from the challenging zone that is in its trajectory before *c*. $p\left({\underline{b}}_{h}^{c-1}:{\underline{a}}_{h}^{c}\right)$ is the path covered by the UAV from the exit point of the previous challenging zone to the its entry point in *c* and $t_{w}\left({\underline{b}}_{h}^{c-1}:{\underline{a}}_{h}^{c}\right)$ is the time required to serve waypoints (if any) during this path. $\bar{v}\left(\;\right)$ defines the mean velocity of the UAV along the path. The entry time is the same for each UAV that serve the *c*-th challenging zone if:

(8) $$t\left({\underline{a}}_{1}^{c}\right)=t\left({\underline{a}}_{2}^{c}\right)=\ldots =t\left({\underline{a}}_{H}^{c}\right),$$

The definition of the time at the entry point of the challenging zone occurs sequentially, therefore once solved the entry time definition at the previous challenging zone, $t\left({\underline{b}}_{h}^{c-1}\right)$ is known with Equation (6), and the only unknowns of the combination of Equations (7) and (8) are the $\bar{v}\left(p\left({\underline{b}}_{h}^{c-1}:{\underline{a}}_{h}^{c}\right)\right)$. They are solved assigning *v_cruise_*/2 to the UAV with the highest $t\left({\underline{b}}_{h}^{c-1}\right)$ and then calculating the velocities for the other UAVs. 

### 5.2. Time of Arrival and Departure for Each Waypoint

The previous step estimates the arrival time at the exit and entry points of the challenging zones, therefore for the *h*-th UAV $t\left({\underline{b}}_{h}^{c}\right)$ and $t\left({\underline{a}}_{h}^{c}\right)$ are known $\forall c=1,\ldots ,c_{h}$, where *c_h_* is the number of challenging areas where the *h*-th UAV passes. This is in general smaller or equal than *C*, i.e., the number of all the challenging areas. As stated in the assumptions, the time of departure from the first waypoint is set as $t\left({\underline{w}}_{h}^{1}\right)=0$. The time of arrival at the last waypoint $t\left({\underline{w}}_{h}^{n_{h}}\right)$ is estimated assuming that after the last challenging zone, the mean velocity of the vehicle is assigned to be equal to *v_cruise_*/2, and the time for flying along the path is defined dividing by the path length (*n_h_* is the number of assigned targets to the *h*-th UAV). Let us call ${\underline{\omega }}_{h}^{i_{\omega }}$ with $i_{\omega }=0,1,\ldots ,2c_{h}+1$, the waypoints for which the arrival time is already known, where ${\underline{\omega }}_{h}^{i_{\omega }=2c-1}={\underline{a}}_{h}^{c}$ and ${\underline{\omega }}_{h}^{i_{\omega }=2c}={\underline{b}}_{h}^{c}$ (note that omega is used instead of w). The sorted sequence of waypoints and exit and entry points of challenging areas is for the *h*-th UAV is:

(9) $$\begin{array}{c} {\tilde{w}}_{h}=\left[\begin{array}{cccccccccccccccc} {\underline{\omega }}_{h}^{0} & \ldots & {\underline{w}}_{h}^{\Delta c_{1}} & {\underline{\omega }}_{h}^{1} & {\underline{w}}_{h}^{\Delta c_{1}+1} & \ldots & {\underline{w}}_{h}^{\Delta c_{2}} & {\underline{\omega }}_{h}^{2} & {\underline{w}}_{h}^{\Delta c_{2}+1} & \ldots & {\underline{w}}_{h}^{\Delta c_{3}} & {\underline{\omega }}_{h}^{3} & \ldots & {\underline{\omega }}_{h}^{2c_{h}} & \ldots & {\underline{\omega }}_{h}^{2c_{h}+1} \end{array}\right]\\ {\underline{\omega }}_{h}^{2c_{h}+1}={\underline{w}}_{h}^{n_{h}},\;{\underline{\omega }}_{h}^{2c_{h}+1}={\underline{w}}_{h}^{1} \end{array}$$

where $\Delta c_{i_{\omega }}$ is the number of waypoints covered by the UAV before the *i_ω_*-th points with already known arrival time. Hence for the *k*-th waypoint, where $\Delta c_{i_{\omega }}+1\leq k\leq \Delta c_{i_{\omega }+1}$, the arrival time is evaluated as:

(10) $$t\left({\underline{w}}_{h}^{k}\right)=t\left({\underline{\omega }}_{h}^{i_{\omega }}\right)+t_{w}\left({\underline{\omega }}_{h}^{i_{\omega }}:{\underline{w}}_{h}^{k-1}\right)+\frac{d\left(p\left({\underline{\omega }}_{h}^{i_{\omega }}:{\underline{w}}_{h}^{k}\right)\right)}{\bar{v}\left(p\left({\underline{\omega }}_{h}^{i_{\omega }}:{\underline{\omega }}_{h}^{i_{\omega }+1}\right)\right)},$$

and the departure time is the arrival time plus $t_{w}\left({\underline{w}}_{h}^{k}\right)$. The mean velocity along the path is obtained with Equations (5) and (7), respectively inside and outside the challenging areas.

## 6. Polynomial Paths

The polygonal trajectory obtained in Section 4 connects the waypoints by polygonal chains and no information about velocity (except for the mean velocity) is available for each segment. To produce smooth trajectories and have a punctual information about the velocity and the acceleration that the UAV is experiencing, polynomial trajectories [37,38] are defined, using for each UAV the assigned waypoints and their time of arrival and departure that are estimated in Section 4 and Section 5.

To obtain the polynomial trajectory this paper uses the method described in [37,38], which results in a UAV path, that is for each position component (x, y and z) a sequence of polynomial segments each of them defined in between two subsequent waypoints. The method described in [37] allows getting a closed-form solution to the quadratic program for polynomial optimization, which aims at minimizing the trajectory snap. The problem can be formulated as linear when the time in between two subsequent waypoints is known, and then easily inverted to obtain the polynomial coefficients [38]. Richter et al. and Burri et al. [37,38] include as problem unknowns not only the polynomial coefficients but also the time to transverse each segment, which turn the problem into a nonlinear optimization problem. In this paper the segment time is strictly dependent on the UAV synchronization performed in Section 5. Hence, the solution of the linear problem gives a polynomial trajectory that passes for the desired waypoint at the desired time epoch. 

Polynomial generation is not only accounting for the waypoint sequence that is defined in (9), but also includes the obstacle avoidance points that are derived in the Section 3. It is assumed that the UAVs fly along the obstacle avoidance waypoints without stopping there. 

**Figure 9:**
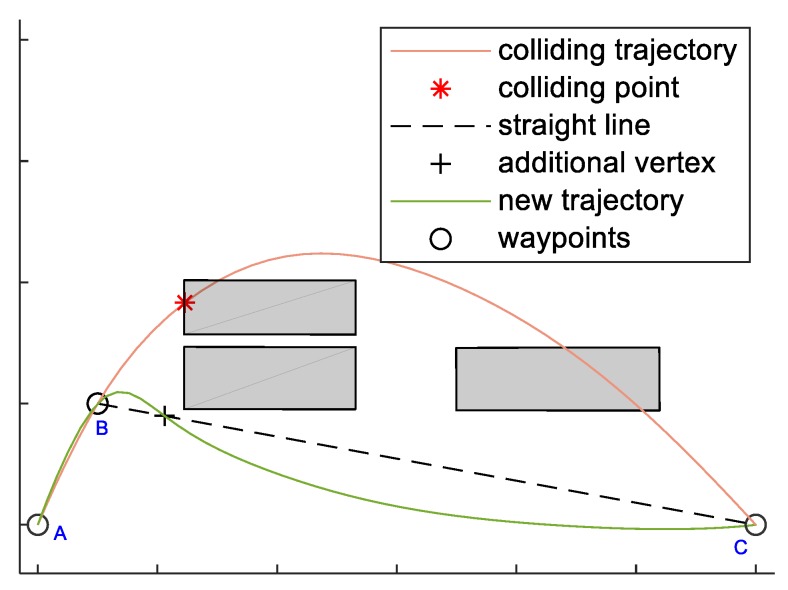
Logic to define an additional vertex to ensure that polynomial path does not collide with obstacles, represented in gray. The polynomial trajectory passing through A, B and C, resulting as linear solution of the polynomial trajectory optimization problem, is the one depicted in orange. This trajectory intersects one of the obstacles (red point). The projection of this point on the straight line (black cross) is computed and the obtained point is added to the original sequence of waypoint between B and C with the proper arrival time.

Using smooth polynomials instead of straight lines does not guarantee that the trajectories are still collision-free. This is handled adding additional vertices on the path in case of collisions. These vertices are computed as projections of the collision points along the polynomial trajectories, see Figure 9. In [38] a similar strategy is adopted, where the new added vertices slow down the trajectory. Due to the need for synchronization among the UAVs, the time of arrival at the new vertices is estimated based on Equation (10) , thus avoiding changes in the arrival times at challenging.

## 7. Performance Assessment

The planning algorithm presented in this paper offers a solution for routing vehicles in an heterogenous environment with the aim of distributing the resources among the targets and using them together when is needed to pass through a challenging area. In this section, the algorithm is tested comparing its performance with those of optimal and heuristic techniques (Section 7.1) and then using it in an applicative example simulating a real-world scenario (Section 7.2). 

### 7.1. Comparison with Optimal and Heuristic Techniques

The target assignment algorithm is tailored to assign the farthest target in order to minimize the overall path length and to equalize paths. In facts, this solution allows obtaining, when the MILP hypotheses are valid, the same results of this optimal technique in terms of targets distribution among the UAVs. This section aims at comparing the performance of our algorithm with optimal techniques, specifically analyzing:

- The classical MILP formulation [16], whose solution is a binary variable $x_{ij}^{h}$ that is 1 if the edge from the target *i* to the target *j* is included in the *h*-th UAV path;
- The MILP formulated as set-covering [16], e.g., Multi-dimensional Multiple-choice Knapsack Problem (MMKP), that instead of the edges assumes the solution for each UAV connected to a circuit, i.e., a feasible sequence of edges. Therefore, the binary variable $y_{l}^{h}$ is 1 if the *l*-th circuit is assigned to the *h*-th UAV, 0 otherwise.

Due to the limitations of the MILP algorithm, a heuristic technique, i.e., Particle Swarm Optimization, has been used to evaluate our algorithm performance for increasing number of targets and UAVs, using the approach described in [32] for discretizing the PSO algorithm.

To allow comparison with MILP, the assignment problem described in the paper has been simplified and no challenging areas have been considered, due to the impossibility of linearizing the formula for father location identification and assignment. In fact, the task assignment algorithm described in this paper is adaptive, and minimizes in central steps of the assignment process, the sum of the distances covered by all the UAVs, while in the first and last steps provides path equalization as reported in Equation (6). Instead, the MILP cost function cannot be tuned adaptively. 

Thus, in this section, first we compare MILP (with and without set covering) and PSO aimed at global distance minimization with a customized version of our algorithm which uses a constant high value for α. Then, we analyze the performance of the proposed algorithm (using the cost function reported in Equation (6) with varying α) in optimizing the overall time, comparing its results with those of the optimal algorithm described in [25]. Performance reported in this section is evaluated on 10 randomly generated scenario, i.e., waypoints location.

**Figure 10:**
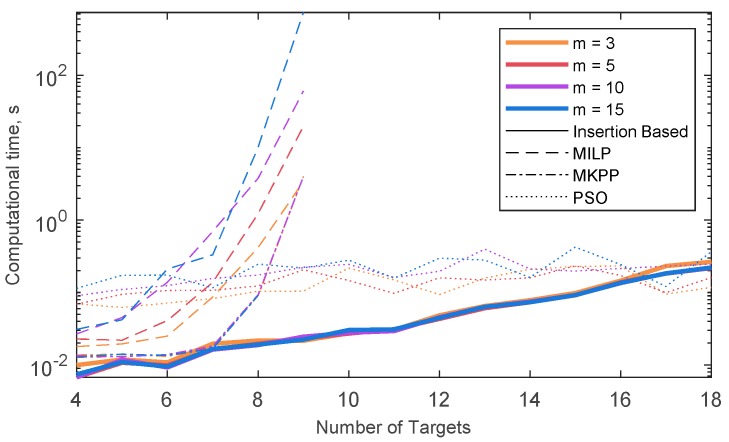
Mean computational time, i.e., running time for obtaining the solution of the VRP, with four different techniques: The method proposed in this paper (Insertion Based), the classical MILP technique (MILP), the MILP formulated as set covering problem (MMKP) and the PSO algorithm. The time is estimated as a mean of the computation time among ten randomly generated sets of waypoints. The edge cost is estimated once for all the techniques and only target assignment time is considered in the picture, to ensure the results are only dependent on the number of targets, and not on the selected scenario. Different number of UAVs (*m*) are used for the simulation identified by different colored lines.

When dealing with overall distance minimization, the solutions of our algorithm (insertion based), classic MILP and set-covering MILP (MMKP), are the same in terms of target assignment, whilst PSO rarely (i.e., only when the number of UAVs and targets is small) yields the optimal solution. Figure 10 shows the mean computation time, i.e., the mean time needed to for the problem to be solved in the ten randomly generated scenarios, for the four different techniques, with different numbers of UAVs. Computational times have been obtained with MATLAB^®^ (The MathWorks, Inc., Natick, MA, USA) on a Windows PC with CPU at 2.20 GHz. In both MILP cases (with and without set covering) the running time is very sensitive to the number of targets, so that no solution is actually available when more than nine targets are considered. In case MILP is formulated as MMKP, the computational burden is not dependent on the number of the UAVs. As for the insertion-based techniques, the computational time is almost independent from the number of UAVs, while it increases for increasing number of targets. However, a reasonably fast solution can be obtained even with a relatively large number of targets. Contrarily, computation time for the PSO solution is strictly dependent on the number of UAVs, and almost constant as a function of the number of targets. To assess the performance of the algorithm in terms of overall time minimization (i.e., “optimality”), it is compared with the MILP algorithm described in [25], that assumes constant velocity. Thus, the overall time minimization can be reduced to minimizing the maximum path length among the UAVs. 

**Figure 11:**
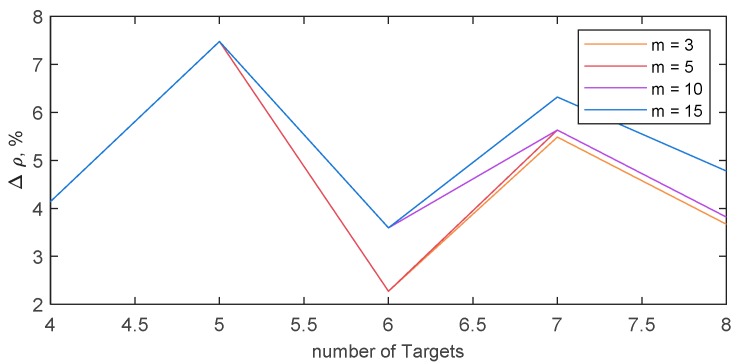
Δρ, i.e., Percentage difference between the maximum path length obtained using the algorithm described in the paper, and the one calculated by an optimal technique aimed at minimizing the maximum path length (and thus the overall mission time if velocity is assumed constant). Δρ is estimated as a mean in ten randomly generated sets of waypoints.

Figure 11 quantifies the capability of our algorithm to mimic optimal overall time minimization, reporting the mean among the 10 randomly generated scenarios of the normalized difference between the maximum path length obtained with our algorithm and with the one described in [25]; i.e., Δρ. Being ν the optimal maximum path length, one can define:

(11) $$\Delta \rho =\frac{\max\left\{d\left(p_{1}\right),d\left(p_{2}\right),\ldots ,d\left(p_{n}\right)\right\}-\nu }{\nu },$$

As far the optimal solution is available, the algorithm proposed in this paper is able to guarantee a maximum path length that is at maximum 8% higher than the optimal. The increment of the maximum path length with respect to the optimal case it is not dependent on the number of UAVs, nor on number of targets.

### 7.2. Results of Routing Algorithm in Real-World Scenario

After analyzing the performance of the proposed algorithm in terms of computational cost, in this section it is tested in a real-world scenario (simplified just neglecting topography variations and considering buildings as prisms). 

#### 7.2.1. Scenario

**Figure 12:**
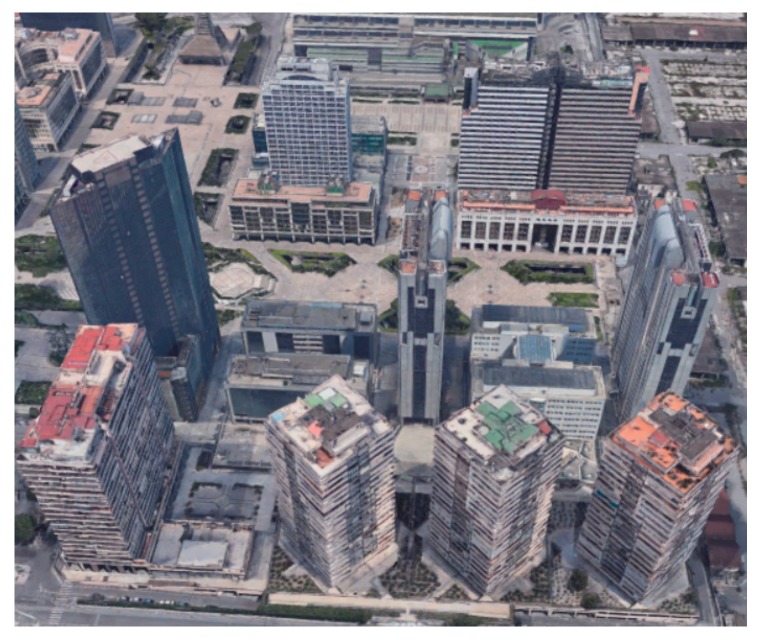
Google Earth’s 3D view of the environment considered for Path Planning, isola C and E of Business district in Naples.

**Figure 13:**
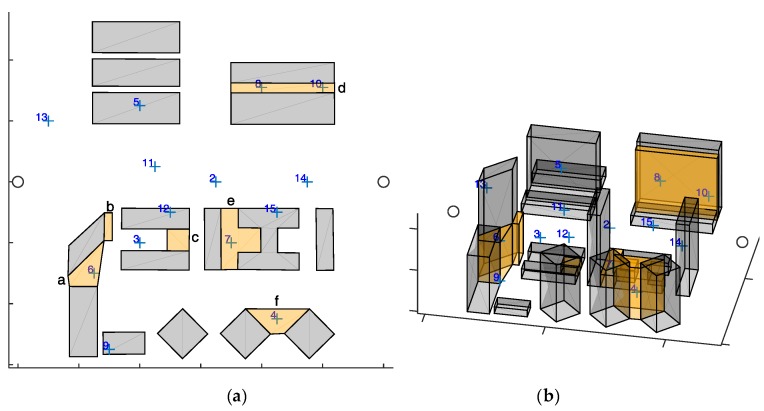
Simulation scenario, (**a**) top view and (**b**) 3D view. Buildings are gray. The Orange areas are GNSS challenging areas. Blue crosses are the targets, whilst gray circles are the starting and ending points that are common to all the UAVs.

The selected scenario is a portion of the Centro Direzionale (Business District) in Naples, i.e., isola C and a portion of isola E. Specifically it is a rectangular region of 300 × 280 m. Within a quasi-unsupervised workflow, the scenario has been imported using freely available 3D maps and commercial software tools, i.e., Open Street Maps (OSM) and Autodesk^®^ Infraworks^®^ and 3ds Max^®^. The 3D representation of the considered scenario in Google Maps is shown in Figure 12, whilst the simulated scenario imported in MATLAB is shown in Figure 13, where the blue crosses represent target waypoints (*n* = 16) and the gray circles identify start and end location.

Buildings and challenging zones are respectively drawn in gray and orange. From the 3D view of the simulation scenario (Figure 13a), it can be noticed that the challenging zones have in general an altitude that is lower than the adjacent buildings. Challenging zones are enumerated with letters. Waypoints 4,6,7,8,10 fall within a challenging area. All the challenging zones contain a waypoint except for *b* and *c*, that are thus seen as obstacles by all the UAVs. In each challenging zone one father is required, except for zone *e* where it is assumed that two fathers are required. Cruise velocity and challenging velocity are respectively *v_cruise_* = 8 m/s and *v_chall_* = 2 m/s, whilst the service time is 0 s for the waypoints outside the challenging, and 1 s for those within these areas.

#### 7.2.2. Results

**Figure 14:**
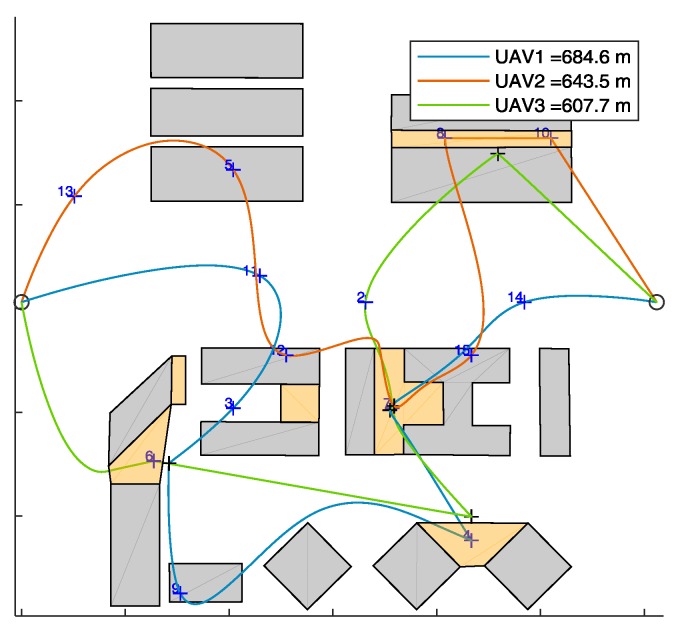
Top view of trajectory generated by the proposed path planning algorithm, for the proposed scenario. *n* = 16, *m* = 3. Father waypoints are highlighted with black crosses. The path length of the UAVs is reported in the legend.

**Figure 15:**
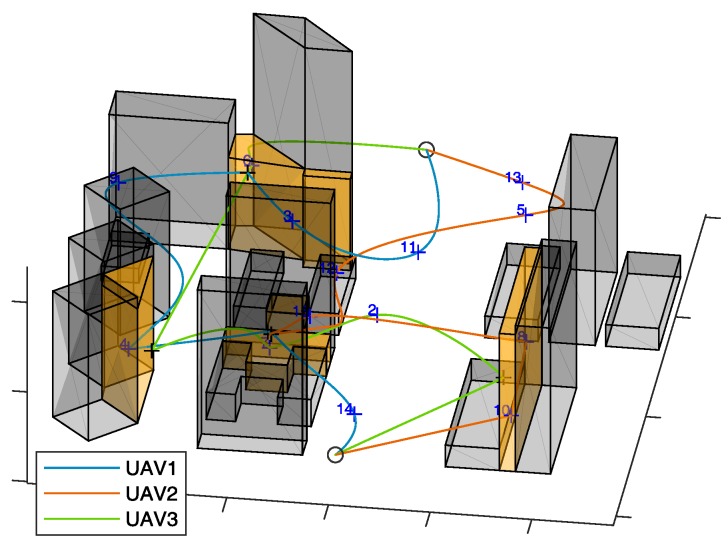
3D view of trajectory generated by the proposed path planning algorithm, for the proposed scenario. *n* = 16, *m*= 3. Father waypoints are highlighted with black crosses.

First, the algorithm has been tested on the selected scenario setting the minimum number of UAVs to fulfill mission requirements. Since zone *e* requires two fathers (and one son), this minimum number is three. UAV trajectories resulting from the algorithm in are shown in Figure 14 (x-y plane), and in Figure 15 (3D). The UAVs paths are smooth due to the usage of polynomial planning. 

**Figure 16:**
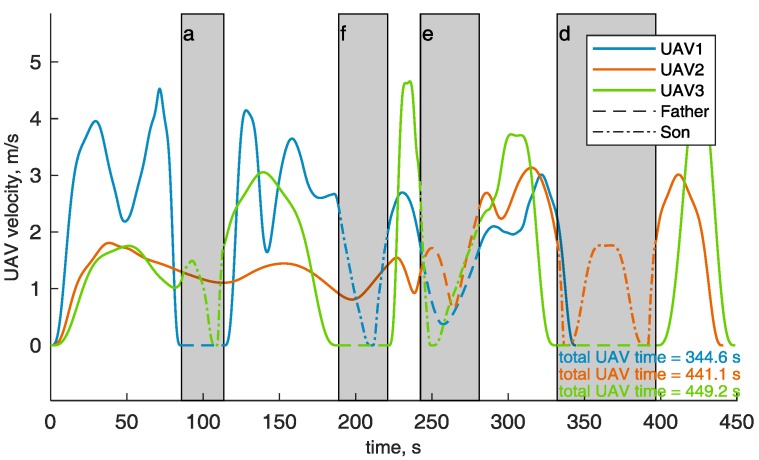
Velocity module of the UAVs during their trajectories, the time to flight along the trajectory is reported in the figure highlight by the corresponding color of the UAV. The challenging area are highlighted by gray background. Note that the challenging areas are sorted in the same order of Table 1. Father and son UAV are highlighted in the challenging area by dashed and dot-dashed lines.

Figure 16 shows the velocities of the UAVs during their paths. In the challenging areas (gray background) the son and father UAVs are highlighted with dashed and dash-dotted lines, respectively. The velocity of the son in the challenging area is always smaller than *v_chall_*, whilst the velocity of the fathers and of all the UAVs outside the challenging area is below *v_cruise_*. The animated version of Figure 14 is available as Supplementary Material (Video S1), and allows the reader visualize the synchronization between the father and the son UAVs, whose times of arrival and departure, reported in Table 1, are the same for each challenging area. Video S1 includes the ROS Gazebo (http://gazebosim.org/tutorials?tut=ros_overview) simulation of the defined trajectories to demonstrate their feasibility, performed with RotorS [39]. The three UAVs are highlighted by sphere of the same colors of the trajectories, centered at the drone centers of mass, and larger than the drone (*r* = 3 m) for the sake of visualization. The mission total duration is about 8 min. Thus, the video is speeded up for the sake of brevity. Table 1 reports the UAVs that are assigned as father and son in the challenging areas, that are sorted in the same order the UAVs pass through them. Challenging areas b and c have no data since there is no waypoint lying there and no UAV is demanded to fly in there. The path length of the three UAVs, reported in Figure 14 is almost the same, as guaranteed by path equalization in Equation (2). As shown in Figure 16, the flight time is shorter for UAV 1 than for UAVs 2 and 3. 

#### 7.2.3. Algorithm’s Performance with Varying *m* and *n*

**Figure 17:**
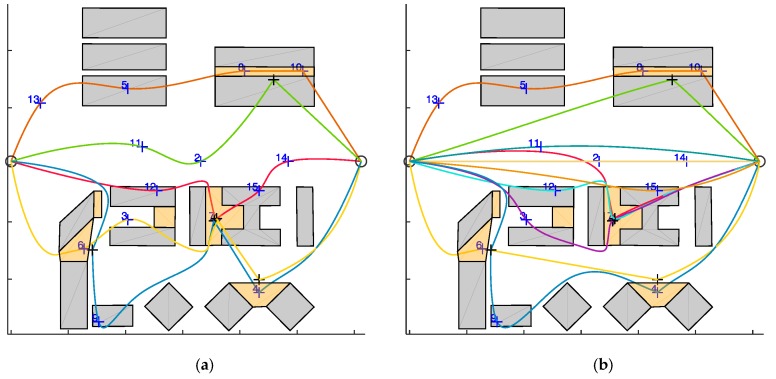
Top view of trajectory generated by the proposed path planning algorithm, for the proposed scenario with *n* = 16 and (**a**) *m* = 5, (**b**) *m* = 11. Black crosses are father waypoints.

**Figure 18:**
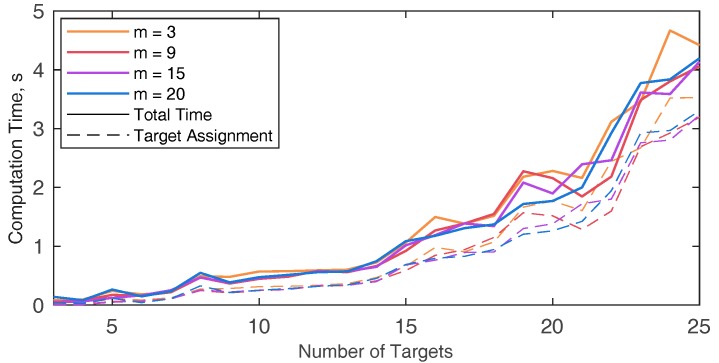
Computation time of the proposed algorithm by varying the number of targets, the total time and the time required for target assignment have been considered. The simulated environment is the one depicted in Figure 13.

The computational burden of the algorithm in the simulated scenario (*n* = 16) with varying number of UAVs is analyzed in Table 2, which reports the computation time needed for each phase of the path planning algorithm along with the total time for running the simulation, as a function of the number of the UAVs (*m*) used to accomplish the mission. The path planning phase that mostly concurs to the computational burden increase is the target assignment. As expected, the computation time is slightly dependent on the number of UAVs used. Indeed, the insertion-based technique used in this paper sequentially adds target to the trajectory and the computation time for each step (i.e., waypoint insertion) is almost constant. Table 3 shows the target sequence assigned to each UAV varying *m* from seven to 20, the UAVs saturation point is obtained at *m* = 11. For the sake of brevity, the targets distribution among the UAVs is reported in Figure 17 only for *m* = 5 and *m* = 11. The only factor that can lead to an increase of computation time is the increase of the number of waypoints, which is analyzed in Figure 18. For the sake of completeness different m values are considered, resulting in a very slight variation of the computational burden when the number of UAVs that composes the fleet varies. The minimum number of targets to assign is equal to 3 since the trajectory is always composed by the start and the end point. The computational burden increases by increasing the number of targets, as expected.

**Figure 19:**
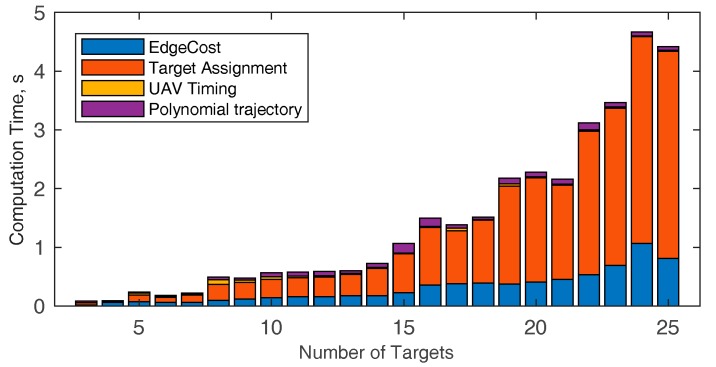
Computation time of the proposed algorithm by varying the number of targets, with *m* = 3. The time required to compute each step of the planning algorithm is stacked in bars, yielding as result the total computation time. The simulated environment is the one depicted in Figure 13.

While Figure 18 reports only the target assignment and the total computation time, the time needed to perform each phase of the algorithm are exploded in Figure 19, when *m* = 3. The increase of the number of targets mainly affects the target assignment time. Polynomial trajectory computation and UAV timing are not affected by the increment of *n*, whilst the edge cost definition rises, due to the increase of the target couples to be considered. The computation time for a larger number of targets (from 25 to 90) is reported in Table 4. The table shows that even with a high number of targets the computation time is compliant with the requirements for pre-mission planning. It could be noticed that in case of UAV failures during the mission, if the number of remaining targets is small (up to 20), the algorithm could be used to re-plan the path, since the running time is compliant with near real time requirements.

For the sake of completeness, Table 2 also shows the mission time, i.e., the maximum flight time among the UAVs. For the analyzed scenario, the mission completion time decreases with the number of UAVs, since path equalization is enhanced by an increasing number of UAVs. Nevertheless, mission time reduction with UAVs number remains constant after *m* = 7. Indeed, even adding more UAVs the minimum path length and thus the minimum travelling time depends on the distance between the start and end point. It is important to notice that for each scenario, (i.e., number of targets, targets location, obstacles and challenging zones) there exists a UAV saturation point, which is the number of UAVs above which, even adding more UAVs to the fleet the target distribution remains the same. In facts, the UAVs beyond the saturation point are not needed for target collection and are demanded only to cover the distance from the start to the end point.

## 8. Conclusions

This paper presented a technique for routing multiple UAVs in a 3D heterogenous environment, characterized by variable coverage of GNSS satellites. In absence of navigation issues, the proposed path planning approach aims at maximizing the efficiency in task assignment by distributing the targets among the UAVs. Instead, in challenging areas, planning allows exploiting cooperative navigation, which is based on the concepts of son and father UAVs. Thus, the multi-UAV fleet is naturally conceived at planning level as a reconfigurable distributed system. 

The complexity of the problem is tackled by a multi-step strategy, including edge definition and cost evaluation, target assignment using a customized insertion-based technique, UAVs timing, and polynomial path definition. The proposed algorithm was tested on a scenario derived from a 3D real-world environment. First, it was shown that when the planning problem is simplified, and optimal techniques are thus applicable, the customized target assignment technique provides the same results of MILP that aims at overall path length minimization, with a lower computational burden. Then, the capability of the proposed algorithm of mimic an optimal solution aimed at mission time minimization was demonstrated. Analyzing the general planning problem, solution behavior for different number of UAVs and targets was investigated. It was demonstrated that the technique provides effective planning solutions taking full advantage from the number of available UAVs, while the computational time is reasonably small even for relatively large number of targets to be covered. 

The presented technique represents a step towards the realization of a fully automated workflow for multi-UAV operations in complex environments. Real time guidance in challenging environments represents another important element that is being addressed in current research.

## Figures and Tables

**Table 1:** Challenging Areas characteristics.

Challenging Zone	Father(s) ID	Son ID	Arrival Time, s	Exit Time, s
a	1	3	24.06	36.35
e	2,3	1	65.80	78.15
f	3	1	90.45	98.96
d	3	2	125.39	151.91
b	-	-	-	-
c	-	-	-	-

**Table 2:** Algorithm performances by varying the number of UAVs used for performing the mission. It is assumed the number of targets is the same defined in used scenario in Section 7.2.1, *n* = 16. The computation time has been estimated for each phase of the planning algorithm. Mission time is the time for mission competition that is the maximum time of flight among the UAVs.

*m*	Computation Time ^1^, s					Mission Time, s
	Edge Cost	Target Assignment	UAV Timing	Polynomial Trajectory	Total Time	
3	0.23	0.78	0.01	0.07	1.09	496.46
4	0.22	0.65	0.01	0.06	0.94	394.27
5	0.23	0.68	0.01	0.08	1.00	333.39
6	0.23	0.72	0.01	0.05	1.01	321.64
7	0.22	0.75	0.01	0.06	1.04	265.74
8	0.23	0.75	0.01	0.06	1.05	265.74
9	0.23	0.75	0.01	0.06	1.05	265.74
10	0.23	0.79	0.01	0.08	1.11	265.74
11	0.22	0.78	0.01	0.08	1.09	265.74
12	0.22	0.80	0.01	0.08	1.11	265.74
13	0.24	0.88	0.01	0.08	1.21	265.74
14	0.23	0.83	0.01	0.08	1.15	265.74
15	0.23	0.83	0.01	0.08	1.15	265.74
16	0.23	0.83	0.01	0.08	1.15	265.74
17	0.23	0.81	0.01	0.08	1.13	265.74
18	0.22	0.83	0.01	0.09	1.15	265.74
19	0.22	0.87	0.01	0.09	1.19	265.74
20	0.22	0.85	0.01	0.08	1.17	265.74

^1^ The computation time has been evaluated with MATLAB running on a Windows PC at 2.2 GHz.

**Table 3:** Target distribution among the UAVs, for a fleet composed by seven to 20 UAVs, *n* = 16. The number of targets with higher index are father targets (target id to 17 to 21).

	Numbers of UAVs (*m*)				
UAV Id	8	9	10	11	11–20
1	1-3-19-9-4-16	1-3-19-9-4-16	1-3-19-9-4-16	1-19-9-4-16	1-19-9-4-16
2	1-13-5-8-10-16	1-13-5-8-10-16	1-13-5-8-10-16	1-13-5-8-10-16	1-13-5-8-10-16
3	1-17-16	1-17-16	1-17-16	1-17-16	1-17-16
4	1-6-18-16	1-6-18-16	1-6-18-16	1-6-18-16	1-6-18-16
5	1-7-15-16	1-7-16	1-7-16	1-7-16	1-7-16
6	1-12-20-16	1-12-2-20-16	1-12-2-20-16	1-12-20-16	1-12-20-16
7	1-3-21-16	1-3-21-16	1-3-21-16	1-3-21-16	1-3-21-16
8	1-11-2-14-16	1-11-14-16	1-11-16	1-11-16	1-11-16
9	-	1-15-16	1-15-16	1-15-16	1-15-16
10	-	-	1-14-16	1-14-16	1-14-16
11	-	-	-	1-2-16	1-2-16
12–20	-	-	-	-	1-16

**Table 4:** Computation Time varying the number of targets and UAVs, simulated scenario is depicted in Figure 13.

		Computation Time, *s*			
	Numbers of UAVs (*m*)	3	9	15	20
**Number of Targets**	30	7.41	7.35	7.18	7.14
	35	12.53	12.39	11.74	11.55
	40	19.04	20.35	20.0	19.83
	50	58.75	60.84	55.35	60.03
	60	109.54	108.90	108.84	108.95
	70	223.12	225.89	224.79	226.57
	80	339.47	336.90	337.45	338.40
	90	594.79	595.54	593.70	596.80

## Supplementary Materials

The following are available online at http://www.mdpi.com/1424-8220/18/12/4188/s1, Video S1: Trajectory of 3 UAVs performing the mission in the simulated scenario (*n* = 16): Gazebo simulation, and trajectories’ top view.
